# Online self-evaluation of fMRI-based neurofeedback performance

**DOI:** 10.1098/rstb.2023.0089

**Published:** 2024-10-21

**Authors:** Santiago Muñoz-Moldes, Anita Tursic, Michael Lührs, Judith Eck, Amaia Benitez Andonegui, Judith Peters, Axel Cleeremans, Rainer Goebel

**Affiliations:** ^1^ Consciousness, Cognition and Computation group, Center for Research in Cognition & Neuroscience, Faculty of Psychology and Education, Université Libre de Bruxelles, Brussels, Belgium; ^2^ Department of Psychology, University of Cambridge, Cambridge, UK; ^3^ Brain Innovation B.V., Research Department, Maastricht, The Netherlands; ^4^ Department of Cognitive Neuroscience, Faculty of Psychology and Neuroscience, Maastricht University, Maastricht, The Netherlands

**Keywords:** neurofeedback, functional magnetic resonance imaging (fMRI), self-regulation, self-evaluation, metacognition, confidence

## Abstract

This study explores the subjective evaluation of supplementary motor area (SMA) regulation performance in a real-time functional magnetic resonance imaging neurofeedback (fMRI-NF) task. In fMRI-NF, people learn how to self-regulate their brain activity by performing mental actions to achieve a certain target level (TL) of blood-oxygen-level-dependent (BOLD) activation. Here, we studied two types of self-evaluation: performance predictions and perceived confidence in the prediction judgement. Participants completed three sessions of SMA regulation in a 7 T fMRI scanner, performing a mental drawing task. During each trial, they modulated their imagery strategy to achieve one of two different levels of SMA activation and reported a performance prediction and their confidence in the prediction before receiving delayed BOLD-activation feedback. Results show that participants’ performance predictions improved with learning throughout the three sessions, and that these improvements were not driven exclusively by their knowledge of previous performance. Confidence reports on the other hand showed no change throughout training and did not correlate with better and worse predictions. In addition to shedding light on mechanisms of internal self-evaluation during neurofeedback training, these results also point to a dissociation between predictions of performance and confidence reports in the presence of feedback.

This article is part of the theme issue ‘Neurofeedback: new territories and neurocognitive mechanisms of endogenous neuromodulation’.

## 1. Introduction

Neurofeedback is a special type of biofeedback that enables self-regulation of one’s brain activity and can be used either by healthy participants aiming to improve their cognitive performance, or as an intervention strategy for symptom improvement in clinical populations (for a review, see [1]). Neurofeedback signals based on real-time functional magnetic resonance imaging (fMRI-NF) offer high regional specificity [2–4] and can thus provide additional information compared with other modalities, such as electroencephalography (EEG) or functional near-infrared spectroscopy (fNIRS) [3,5,6]. Especially high field (7 T) MRI provides a high signal-to-noise ratio [7]. This can be particularly beneficial during gradual, level-specific self-regulation of the blood-oxygen-level-dependent (BOLD) signal [8–10], by providing more degrees of freedom for learning self-regulation in the context of neurofeedback and brain–computer interfaces (BCI) than more conventional up- or down-regulation.

Neurofeedback demonstrates the potential as a method for training self-regulation of brain activity (for reviews see [11,12]), but the specific mechanisms by which individuals learn this skill remain unclear. The type of neurofeedback protocol and the instructions provided likely play a key role [1,2,13,14], with theories proposing mechanisms that range from instrumental conditioning [15–17] to enhanced awareness of internal states [18–21]. One crucial factor in the effectiveness of neurofeedback appears to be self-evaluation, represented by an internal estimation of one’s own performance. This internal performance estimate seems critical for successful performance in neurofeedback transfer trials, where individuals attempt to regulate their brain activity without real-time feedback. Several studies indeed reported successful regulation during transfer runs [22–24], including regulation to different levels [10], but none investigated how self-evaluation contributed to this success. Understanding how participants evaluate their brain activity during neurofeedback could be essential for optimizing training and improving its clinical applications. Research focusing on strategies to enhance the accuracy of this process could make significant contributions to the field of neurofeedback.

One crucial component of neurofeedback self-regulation is metacognition. Metacognition—cognition about cognition—can be defined as the self-evaluation of the quality of neuronal evidence [25,26]. It is associated with performance evaluation and error awareness, two different, but interdependent processes [27,28]. In the case of neurofeedback and BCI, metacognitive decisions can be understood as a form of internal evaluation detached from somatosensory feedback, a process that is thus different from the evaluation of executed movements [29]. In neurofeedback tasks, two types of self-evaluation can be distinguished: the evaluation of a mental action or signal (e.g. the prediction of the feedback value), and the evaluation of one’s own evaluation (i.e. an assessment of one’s own self-evaluation performance, such as the confidence of having made an accurate prediction of the feedback). As neurofeedback often requires practice over multiple sessions [30], it can be considered a suitable candidate for studying changes in performance prediction and confidence over the course of neurofeedback learning.

In the present work, we study how performance prediction for self-generated mental actions and its associated confidence evolve during neurofeedback-guided motor imagery training. Participants were trained to adjust their motor imagery to reach one of the two pre-established target levels (TLs) of supplementary motor area (SMA) activity. The intensity levels were defined as a function of the maximum self-regulation performance in an initial fMRI localizer task. Importantly, participants expressed interleaved performance predictions (i.e. predictions of feedback) and associated judgments of confidence, before receiving intermittent neurofeedback from the SMA region using a 7 T MRI. We hypothesized that as neurofeedback performance improves, participants would increase their accuracy in their self-evaluations, as evidenced both by a higher performance prediction accuracy, and a higher match between confidence and prediction accuracy.

## 2. Methods

### (a) Participants

Eleven participants with no prior neurofeedback experience were recruited at Maastricht University (Maastricht, The Netherlands) to undergo five training sessions, one per day and all completed within 11 days. One participant performed an alternative version of the task with other experimental parameters (P01), another did not finish all sessions (P03) and a third failed to follow the instructions of the task (P10); we thus excluded them from further analysis. The final sample consisted of eight healthy volunteers (four females), aged 25−32 years (*M* = 27.5, s.d. = 2.5), all right-handed, with normal or corrected-to-normal vision and without any history of psychiatric or neurological disorders. Note that the original participant labels (P01–P11) were kept for consistency. Participants provided informed consent and received financial compensation for taking part in the study.

### (b) General procedure

The experimental procedure was approved by the Ethics Review Committee Psychology and Neuroscience at Maastricht University. The study consisted of five sessions, during which participants completed trials of a motor imagery task interleaved with self-reports of performance prediction and confidence (see §2c for details). The first and fifth session took place with a fNIRS measure outside the scanner and without neurofeedback. The other three sessions (second, third and fourth) were performed in the 7 T fMRI scanner and included intermittent neurofeedback after every trial. Here, we only present data of the neurofeedback fMRI sessions (second to fourth session), hereafter referred to as sessions first, second and third.

Each fMRI session lasted approximately 2 h and consisted of one anatomical measurement and seven functional runs. The first functional run was an 8 min localizer scan used to define the target region and the signal change for the neurofeedback runs. The subsequent neurofeedback runs lasted 9 min each and included 60 trials of self-regulation (10 trials per run). Hence, each participant performed a total of 180 trials (90 per condition) across the three sessions.

Participants were informed about the principles of neurofeedback and that their goal in the experiment was to ‘learn how to achieve different levels of brain activation by modulating a mental drawing task’. They were given suggestions for cognitive strategies (see electronic supplementary material for details).

### (c) Tasks

#### (i) Functional localizer task

The localizer run consisted of alternating 16 s blocks of mental drawing, finger tapping and rest to define the target region and the thresholds for the neurofeedback task (see §2e(ii)). Participants completed eight blocks of drawing, eight blocks of tapping and 17 blocks of rest.

#### (ii) Motor imagery neurofeedback task

**Figure 1.**
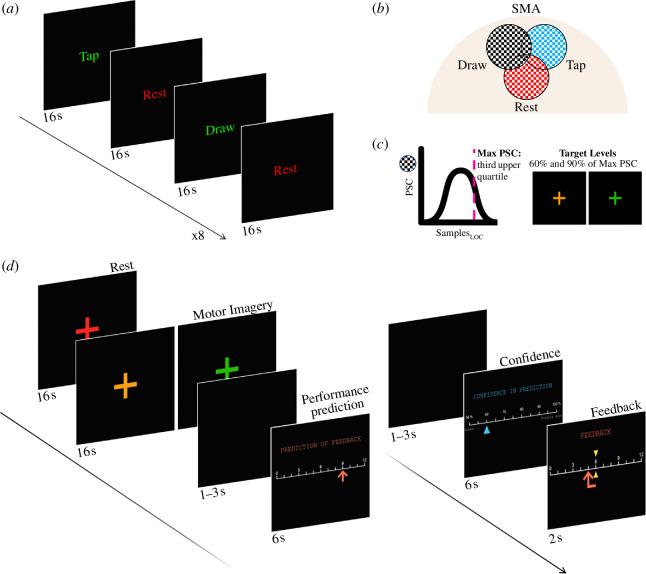
(*a*) Functional localizer task. (*b*) Target region of interest (ROI) selection. (*c*) Maximum percent signal change (MaxPSC) was defined by taking the third upper quartile value of PSC. Two target levels (TLs) for the neurofeedback task were defined as 60% (orange) and 90% (green) of the MaxPSC. (*d*) Neurofeedback runs included self-ratings of the: (i) performed regulation level (‘performance prediction’) and the (ii) confidence of this performance prediction (‘confidence in prediction’), followed by the feedback of the achieved regulation level (orange arrow) and its difference to the TL (yellow marker).

Stimuli were presented using the Expyriment package (v. 0.9.0) for Python (v. 2.7.10) [31]. Each trial started with a red cross signalling the rest period and the spoken word ‘rest’. After 16 s, the motor imagery period started with a change in colour of the cross (to yellow or green) and a simultaneous auditory cue (‘six’ or ‘nine’), indicating the TL to be achieved through motor imagery, 60% or 90% of the maximum per cent-signal change (MaxPSC) in the functional localizer. The order of TLs was pseudorandomized so that half of the trials in the run requested the participants to regulate to level 60% and half to level 90%. After 16 s of continuous motor imagery, an auditory cue ‘stop’ indicated the end of the motor imagery trial. This was followed by a jittered blank screen of 1-, 2- or 3 s duration. Participants were then shown a horizontal rating scale showing values from 0 to 12, with 6 and 9 representing the two TLs (60% and 90%, respectively). They were asked to report their performance prediction by moving left or right on the scale with two buttons of the button box. After another jittered blank screen (1, 2 or 3 s), a second scale was presented, and participants were asked to report their confidence in their prediction. The horizontal scale ranged from 50 to 100% increasing in steps of 5%, with two labels on the end points, indicating ‘guess’ and ‘totally sure’. For both scales, participants had 6 s to respond, and the start position of the cursor was jittered around the midpoint to prevent motor preparation. Participants were then shown the neurofeedback value on the same 13-point scale for 2 s, with an arrow pointing from the TL to the achieved value regulation (see figure 1*d*).

*Instructions for ratings*. For self-ratings of the performed regulation level (performance prediction), participants were asked to rate the average level of activation that they thought they had achieved during the imagery period (or, in other words, to predict the neurofeedback value they would obtain for the current trial). For confidence ratings, participants were asked to rate their confidence in their predicted performance. They were reminded not to confuse this with confidence in having reached the TL or having performed better or worse. Here, we insisted that the question was about the confidence in the performance prediction.

*Control trials*. In each neurofeedback run, 1 of 10 trials was a catch trial in which the responses for the performance prediction and confidence rating were instructed. In these control trials, two red bars surrounded one of the values on the scale and participants were asked to move the cursor to the indicated location. These catch trials were included as an attention check, but also to reduce the confounding effect of the motor movement in the judgement process [32] during offline analysis.

### (d) Data acquisition

MR images were recorded using a Siemens Magnetom 7 T MR scanner with a 32-channel head coil. Anatomical images were acquired with a T1-weighted MP2RAGE sequence; 256 sagittal slices, voxel size = 0.9 × 0.9 × 0.9 mm^3^. Functional images were obtained using a gradient echo (T2*-weighted) echo-planar imaging sequence, with the following parameters: echo time = 21 ms, repetition time = 1000 ms, multi-band factor = 3, flip angle = 60°, matrix = 224 × 224, number of slices = 60, voxel size = 2 × 2 × 2 mm^3^. The field of view provided almost whole-brain coverage.

Behavioural responses were recorded with a fibre optic four-button response box (Current Designs, https://www.curdes.com) attached to the participants’ right hand with the index and middle finger placed on buttons 1 and 2, respectively.

### (e) Online analysis

#### (i) Preprocessing

MR images were reconstructed in real time and exported to a dedicated computer, via a direct transmission control protocol/internet protocol (TCP/IP) connection, where they were preprocessed using Turbo-BrainVoyager (v. 3.2, Brain Innovation B.V., Maastricht, The Netherlands). Each functional volume was motion corrected to the first volume of the functional localizer run of the same session and spatially smoothed using a Gaussian kernel of 4 mm. Linear trends were modelled as an additional predictor in the general linear model (GLM; see §2e(ii)). The real-time processing computer and the stimulation application communicated over the network using a direct TCP/IP connection.

#### (ii) Target region selection

The neurofeedback target region was defined for each participant in each session separately, combining expert knowledge and an algorithm for automated selection of brain areas [33] based on a GLM analysis of the functional localizer data. Since activation related to imagined movement is well predicted by the SMA [34], the anatomical scan was used to preselect an area that corresponded to the location of the SMA; this area was then further restricted by using the functional localizer task (figure 1*a*). The resulting area comprised voxels that were more active for mental drawing than for real movement and rest. Within this area, the algorithm automatically selected the final target region: 30 most significant voxels, forming one contiguous cluster with a 26 neighbour-voxel criterion spanning over not more than six contiguous slices.

#### (iii) Calculation of neurofeedback

The localizer data were used to calculate individualized MaxPSC for mental drawing blocks, for each session of each participant. To account for potential future fatigue, each MaxPSC was determined by calculating the third upper quartile (rather than maximum) of average mental drawing per cent signal change (PSC).

Intermittent neurofeedback was then calculated as the PSC value during each mental drawing trial with respect to its preceding baseline window (equation 2.1). To account for the BOLD delay, only the last few volumes of each rest and mental drawing period were considered. The baseline value corresponded to the mean activation between −4 and +2 s with respect to imagery block onset (six volumes), whereas the imagery value was the average from +6 to +16 seconds (10 volumes). The PSC of each trial was then divided by the participant’s MaxPSC and multiplied by 10 to obtain a normalized value where 10 corresponded to the MaxPSC (equation 2.2).


(2.1) $$PSCnf=\;\frac{mean\;imagery\;-\;mean\;baseline}{mean\;baseline}\;\times 100,$$

(2.2) $$Neurofeedback=\;\frac{PSCnf}{MaxPSC}\;\times 10.$$

The neurofeedback and the performance prediction scale presented to the participants included values from 0 to 12 (instead of 0–10, to allow the presentation of values above MaxPSC). Consequently, the participants were aware of a potential overshoot when regulating, which allowed them to further improve the learning process. Twelve was chosen specifically to equalize the information range between the TLs 60% and 90%, and the value presented maximally on the scale. Values below 0 and above 12 were clipped to ‘0’ and ‘12’, respectively.

### (f) Offline analysis

To estimate how the main outcomes (i.e. self-regulation performance, performance predictions and confidence reports) differed according to experimental conditions, we used R (v. 4.0) [35], Stan (rstan v. 2.16) and the brms package (Bayesian Regression Models using Stan v. 2.1.) to fit multi-level Bayesian linear models. The use of multi-level modelling allowed us to estimate the effects of interest for each participant individually [36]. The use of the Bayesian framework of brms over maximum-likelihood-based approaches to multi-level modelling provided several benefits, such as the improved rates of convergence, the ability to make direct probability statements and the obtention of more intuitive uncertainty estimates than those of null-hypothesis significance testing [37].

All three models of the main outcomes were estimated with Markov chain Monte Carlo sampling, running two parallel chains for 5 000 iterations each (the first 2 000 warm-up samples for each chain were discarded). For each model, we assigned random slopes and intercepts for individuals [38], while priors were kept to default. We report posterior means and credible intervals [37,39]. The posterior probability distributions from the model parameters were also used to test several hypotheses, which are listed in the subsequent sections. Since the hypothesis() function of the brms package does not allow for computing evidence ratios when using default priors, these hypotheses were formulated as one-directional. For each hypothesis, we therefore computed the posterior probability of the hypothesis against its alternative (for our one-directional hypotheses, this quantity corresponds to the proportion of the posterior probability above 0). The formulation of several of the one-directional effects was driven by the observations during real-time sessions and by preliminary results, so rather than *a priori* hypotheses, these should be seen as statements that guide the exploration and visualization of the results. Each hypothesis test was applied to each individual.

For the remaining statistical analyses, null-hypothesis significance testing was used in R (v. 4.0) [35] for the pointwise *t*-tests in the within-trial PSC time-course analysis (§2f(i)) and MATLAB (R2018b, MathWorks, Natick, MA) for ANOVAs. If not stated otherwise, *t*-tests and ANOVAs were carried out two-sided and with the alpha threshold level of 0.05.

#### (i) Percent-signal changes in target region of interest

**Figure 2.**
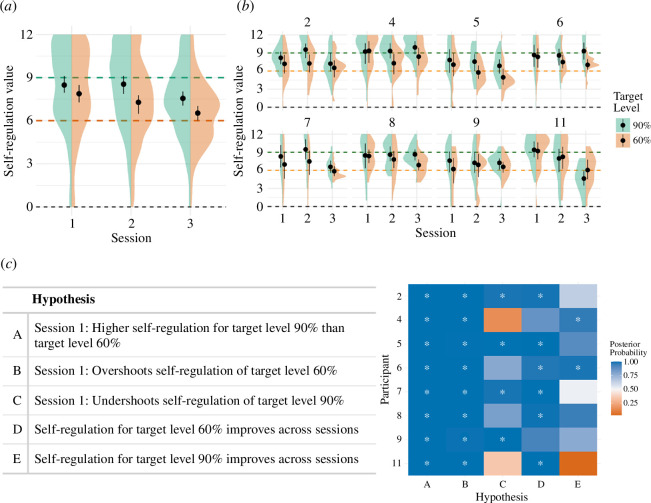
Self-regulation performance. (*a*) Group average for self-regulation performance. For each session (1, 2 and 3) and TL (60% or 90%, 6 or 9 on the 0–12 scale), the mean and within-subjects confidence intervals are shown. In colour, the probability density distribution of the underlying data is shown, trimmed to the range of the data. (*b*) Similar to *a*, separated for each participant. (*c*) Table showing the posterior probability value for each hypothesis statement tested using the self-regulation model. The posterior probability corresponds to the proportion of samples from the posterior distribution of the parameters conforming to the hypothesis. A value above 0.5 (50%) indicates a higher proportion of samples in agreement with the hypothesis and is illustrated with the fill colour (from red = 0%, over white = 50%, to blue = 100%). Asterisks indicate a posterior probability that exceeds 95%. Note that the participant identifiers equal the initial numbering (before exclusion).

*Whole-trial PSC*. To find out whether neurofeedback self-regulation performance (i.e. how far the achieved self-regulation deviated from the TL of 60% or 90%) improved across sessions, we modelled the self-regulation outcome (centred around the TL) with the TL (60% or 90%) and the Session (1, 2 or 3), as predictors. We tested five hypotheses. First, we asked whether participants achieved a higher activation for level 90% than for level 60%. We also checked whether participants undershot when trying to reach level 90% or overshot when trying to reach level 60%. In the final two hypotheses, we investigated whether the participants improved across sessions for either level. All five hypotheses are presented in figure 2.

#### (ii) Performance prediction

**Figure 3.**
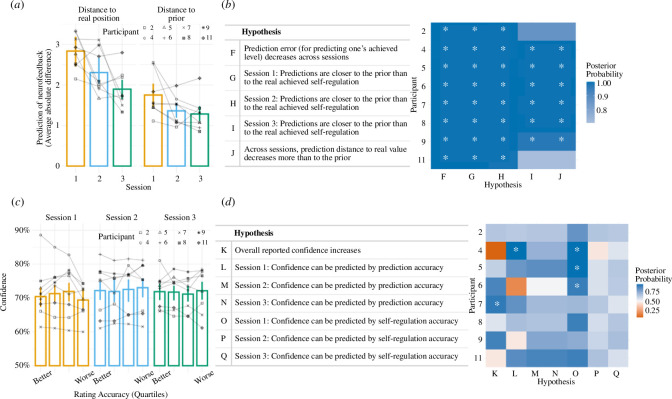
(*a*) Average performance prediction accuracy per session. Left panel: distance to real position (i.e. the absolute difference in each trial between the participant’s prediction and the self-regulation value). Right panel: distance to the prior (i.e. the absolute difference in each trial between the participant’s prediction, and a prior consisting of the running average of self-regulation achieved in the previous five trials). The connected shapes represent each individual participant. (*b*) Individual results of hypothesis testing for performance prediction. Table showing the posterior probability value for each hypothesis statement tested using the performance prediction model. (*c*) Trial-by-trial confidence ratings as a function of performance prediction accuracy (performance prediction accuracy—‘rating accuracy’ —has been split into quartiles for illustrative purposes only). The connected shapes represent each individual participant. (*d*) Individual results of hypothesis testing. Table showing the posterior probability value for each hypothesis statement tested using the confidence model. Note that the posterior probability corresponds to the proportion of samples from the posterior distribution of the parameters conforming to the hypothesis. A value above 0.5 (50%) indicates a higher proportion of samples in agreement with the hypothesis and is illustrated with the fill colour (from red = 0% over white = 50% to blue = 100%). Asterisks indicate a posterior probability that exceeds 95%. Participant identifiers equal the initial numbering (before exclusion).

We analysed whether predictions of self-regulation performance became more accurate across sessions (i.e. the prediction moved closer to the actual achieved neurofeedback value). Since we hypothesized that performance predictions could also be driven by feedback received in previous trials, we controlled for this possibility by calculating the running average of performance as the average neurofeedback obtained in the last five trials of the corresponding TL and modelled the performance prediction using *target reference* (real position versus prior of previous performance) and *session* (1, 2 or 3) as predictors. From the model results, we tested five hypotheses, see figure 3*b*. We wanted to see whether the prediction error decreases across sessions. Then, we tested whether the prediction is closer to the prior (values) or real achieved values for each session separately. Finally, we tested whether the distance between the prediction and real value decreased more than the distance between the prediction and prior.

#### (iii) Confidence in prediction

To investigate whether reported confidence changed throughout sessions and whether participants developed the ability to differentiate between their better and worse performance predictions, we modelled the confidence report on the *performance prediction accuracy* (the absolute difference between the performance prediction and the neurofeedback value), the *session* (1, 2 or 3) and the *self-regulation* value (0–12). The self-regulation was added to account for the possibility that confidence would be influenced by the self-regulation level achieved, even though participants were asked to report their confidence that their prediction was correct. From the model results, we tested the seven hypotheses presented in figure 3*d*. We first wanted to find out if confidence increases over sessions. Next, we aimed at exploring whether confidence reports can be predicted by the prediction accuracy or by self-regulation accuracy. This was tested for each session separately.

## 3. Results

### (a) Per cent signal change in target region of interest

#### (i) Trial-by-trial self-regulation

In the following section, group results are described by the group posterior means (on the 0–12 self-regulation scale) with their 95% confidence interval. In the first session, on average, participants achieved a 0.61 [0.16–1.06] higher self-regulation for TL 90% than TL 60%. At the individual level, this was the case for all participants (*hypothesis A*). In the first session, for TL 60%, participants reached an average of 7.86 [7.2–8.55], which was above the target. At the individual level, all participants overshot the target value (*hypothesis B*). In the first session, for TL 90%, participants reached an average of 8.47 [7.91–9.01], which was below the target. At the individual level, four participants undershot compared with the target value (*hypothesis C*). Regarding learning (difference between session 1 and session 3), results show that, on average, self-regulation for TL 60% improved, as the average self-regulation level decreased for 1.32 [0.58–2.06], from 7.86 to 6.54, and therefore moved closer to the TL of 60%. At the individual level, the improvement was visible for six out of eight participants (*hypothesis D*). Learning effects for TL 90%, however, were less clear, as the average regulation increased by 0.42 [−0.57–1.42] (the range includes 0) and therefore moved closer to the TL of 90%, but individually, the improvement was only noticeable for two participants (*hypothesis E*). See electronic supplementary material for the model summary and the list of hypothesis formulae.

### (b) Predictions of performance

In the following section, group results are described by the group posterior means (on the 0–12 neurofeedback scale) with their 95% confidence interval. The results from the model show that prediction error (i.e. the absolute difference between the prediction and achieved level) decreases across sessions. On average, participants’ prediction error was 2.81 [2.45-3.16] in session 1 and 1.87 [1.39–2.35] in session 3, meaning that the prediction became 0.94 [0.46–1.41] points closer to the actual achieved value. At the individual level, all participants showed a decrease in prediction error across sessions (*hypothesis F*). When looking at the potential influence of knowledge of previous performance, results show that, for all sessions, participants’ predictions are closer to the running average of previous performance than to the real achieved self-regulation in the trial. At the individual level, this was the case for all participants except for two in the last session (*hypotheses G, H* and *I*). We also investigated whether previous performance could explain the improvements in performance prediction accuracy: while prediction error with respect to the prior also decreased, the decrease with respect to the real position was higher. This indicates that participants’ performance predictions became closer to the real position and that changes in prior do not account for this difference entirely. Alternatively, it could be argued that the learning effect is due to the statistical principle of the regression of the mean, explaining a general reduction in prediction error over time. However, we contend that this explanation falls short in clarifying the observed differential effect in figure 3*b*—*hypothesis J*, where the prediction error to the real (self-regulation) value decreases more over time than the prediction error to the prior. This discrepancy is better explained for by a true learning effect. The distance to real position decreased by 0.16 [−0.31–0.62] in session 2, and by 0.53 [0.88–0.17] in session 3 more than did the distance to the prior (*hypothesis J*). See electronic supplementary material for the model summary and the list of hypothesis formulae.

### (c) Confidence in predictions

In the following section, group results are described by the group posterior means (on the 50%–100% confidence scale) with their 95% confidence interval. The results show that on average, reported confidence for performance predictions did not increase across sessions (with an average confidence of 69.95% [63.1–75.6%] in session 1 and confidence of 70.5% [64.1–76.45%] in session 3). At the individual level, indeed, an increase in confidence was only noticeable in one participant (*hypothesis K*). Additionally, we found that confidence did not depend on the accuracy of the predictions, in either session. That is, confidence levels did not differ between the best and worst predictions of performance (session 1: 0.06 [−0.17–0.05], session 2: 0.06 [−0.08–0.19], session 3: 0.03 [−0.12–0.19]). Looking at individual differences, only one participant in session 1 showed an effect of performance prediction accuracy on confidence, but this effect was not present in the following sessions (*hypotheses L, M, N*). Lastly, while confidence was affected by the self-regulation level achieved in three participants in session 1, the effect for those three disappeared in the following sessions as well, and other participants did not show any effect of self-regulation performance in either session (session 1: 0.06 [0.00–0.13], session 2: −0.01 [-0.08–0.06], session 3: −0.02 [-0.10–0.07]) (*hypotheses O, P, Q*). See electronic supplementary material for the model summary and the list of hypothesis formulae.

## 4. Discussion

The capacity to monitor our ongoing mental activity is an important component of mental self-regulation, and yet its role in neurofeedback and BCI learning remains largely unaddressed. Here, we measured people’s capacity to self-regulate the activity of a target brain region and to evaluate the level of activation they achieved and their confidence in their estimation, while receiving intermittent neurofeedback information. Intermittent feedback was crucial to gather subjective self-reports before participants were informed about their actual performance. We revealed evidence for an improvement in self-evaluation of mental self-regulation, confirming our hypothesis that neurofeedback guides the enhancement of predictions of performance. However, the pattern of responses we observed for confidence reports invalidated our other hypothesis: although self-regulation performance and performance predictions improved, confidence did not change and was not diagnostic of performance prediction accuracy. We separately discuss the results for self-regulation, predictions of performance and confidence.

### (a) Self-regulation improves with learning

Participants were asked to self-regulate their brain activity to one of two TLs of their individual SMA activity. The results showed that training improved the participants’ ability to self-regulate to different TLs, which is in agreement with previous studies [8,9,40]. Participants were already relatively close to reaching level 90% in the first session, perhaps due to its proximity to MaxPSC; the regulation improvement was therefore particularly clear for level 60%. A potential explanation would be that reaching different TLs is more difficult using intermittent feedback as compared with the continuous feedback approach employed by earlier studies [8,9,40]. More neurofeedback sessions should be employed in future studies and might help to achieve a more robust TL regulation.

### (b) Participants improve their performance prediction

After each trial, participants were asked to evaluate their performance by providing a performance prediction for that trial. We found that although participants estimated their performance more accurately when they also performed better (i.e. their regulation performance more closely matched the actual TL), they did not necessarily rely solely on their self-evaluation. Crucially, participants’ performance predictions improved throughout training, although they remained closer to their previous performance than to the real achieved values in each trial. However, relying on heuristics based on previous performance is insufficient to explain all improvements in prediction accuracy. Indeed, our results showed that prediction-errors with respect to the real position decreased more than prediction-errors with respect to the prior, indicating that at least part of the improvements in the prediction-error are not explained by the prior heuristic.

Evaluating performance in the context of neurofeedback can be particularly difficult. In many studies of performance self-evaluation or metacognition, the object of evaluation is typically a form of exogenously evoked signal (e.g. as in confidence in visual perception) and is often accompanied by motor signals. People hence have access to several multi-sensory cues (sensory, motor, etc.) that can be integrated to inform their self-evaluation of performance [41,42]. Because here the signal to be evaluated was self-generated, somatosensory afferents were absent or irrelevant. We speculate that this aspect inevitably led participants to use heuristics for their performance predictions [29,43].

As participants performed multiple trials, a heuristic for their estimates became their previous performance. We found that as participants’ predictions became more accurate with learning, the use of the heuristic diminished. This diminution in the use of the heuristic is logically derived from the finding that prediction errors decreased across sessions, and, importantly, that this decrease in errors could not solely be attributed to the decrease in error with respect to the prior. Future studies could take advantage of using transfer runs or sham groups to investigate the effects of this heuristic on the performance prediction by not providing any feedback or incorrect feedback, respectively.

Our conclusion aligns with Schurger *et al*. [29], who observed that participants learned to better evaluate their actions with EEG-based motor imagery through training. They are also in line with previous EEG-based neurofeedback studies looking at self-discrimination of the alpha rhythm [16,20,44]. Here, using fMRI-NF, we further show that the self-evaluation capacity can be achieved for mental actions targeting the self-regulation of a circumscribed brain region.

### (c) Is confidence a reliable index of prediction accuracy?

Our results contribute new insights into the relationship between confidence and the accuracy of performance predictions, highlighting the concept of confidence sensitivity. However, further research is needed to fully elucidate this complex relationship. Previous studies looked at the capacity to discriminate or monitor mental actions, using only evaluations of performance. Here, we included an additional judgement layer, the judgement of the quality of one’s own prediction accuracy, by which we aimed to measure the participants’ ability to differentiate between simple guessing and informed judgements. We found that, although not all performance predictions were equally accurate, confidence did not differentiate between the better and the worse ones.

There are multiple ways in which confidence can relate to performance. A normative view is that confidence is a subjective probability and it is based on the probability that a choice that one made (e.g. a prediction in our case) was correct given the evidence [45–48]. But confidence can also be driven more directly by characteristics of the signal itself, such as its perceived uncertainty [49] or the magnitude of sensory data [47,50]. To illustrate based on our task, envision a signal generated by a participant in SMA, measured in real-time as a sample of BOLD PSC values with mean *M* and variance *V*. An ideal observer would respond, based on our instructions for the task, as close to *M* as possible on the objective scale, and give a confidence report that takes into account how close (accurate) they were in their prediction (e.g. higher confidence for smaller errors). Alternatively, someone might provide a simpler confidence estimate that depends on the signal’s uncertainty (*V*), like its inverse (1 /*V* for the confidence scale, where confidence is inversely related to variance; lower confidence for higher variance and vice versa).

Other factors can also contribute to confidence. Here, MaxPSC was adjusted which caused an implicit adjustment of the TLs for each session, making the present neurofeedback task rather difficult. Overall task difficulty, for instance, is indeed an important contributor to confidence [51] and also to participants’ perceived performance ranking in the group; difficult tasks tend to make good (or experienced) performers underestimate their performance and make bad performers overestimate it [52,53]. On the other hand, tasks resulting in highest performance accuracy also resulted in the lowest confidence reports, with little difference in confidence between correct and incorrect answers [54]. Taking these results into consideration, we would therefore expect that the participants who predicted their performance well would rate their confidence as rather low relative to their performance; the remaining participants would misjudge their performance more, but with more confidence than their performance would suggest. The convergence of the confidence reports with training seems to be in line with this theory, especially given the difficulty of the present task and the lack of confidence improvement even when provided with feedback.

Subsequent studies should investigate how various factors impact confidence, including difficulty, error rates in prior trials, expectations about progress and more. Moreover, incentivizing participants based on the accuracy of their confidence judgments (as demonstrated by [55]) may enhance the alignment between confidence and performance. As we acknowledge the limitations of our current study, characterized by its sample size and experimental design, we advocate for future research to expand participant and trial numbers, thereby facilitating a more comprehensive and conclusive examination of the confidence dynamics.

### (d) Potential limitations

Several potential limiting factors exist in the current study. First, there is a limited number of participants (*n* = 8), which in principle can result in lower statistical power to detect the hypothesized effects and higher sensitivity to outliers in the sample. However, all analyses were conducted within participants across multiple training sessions, yielding consistent results. We also note that the use of ultra-high field (7 T) fMRI is associated with improvements in the signal-to-noise ratio (up to 200%−300% when compared with 3 T), thus also increasing power for statistical sensitivity [7,56].

Second, we were unable to measure muscular activity in the hand. Although movement was visually monitored during the initial training session outside of the scanner, it is still possible that participants relied (unconsciously) on sub-threshold muscular activity to perform self-regulation of the target region. However, a prior study did not find electromyographic activation driving motor imagery [57]. Furthermore, our ROI selection partially controlled for overt movement by choosing voxels with higher activation for mental imagery than finger tapping.

Last, to mitigate the impact of prior performance knowledge on predictions, we employed the running average of the five preceding trials. This choice was made to allow for the running average to reflect not only longer, but also shorter time-scale variations in performance. However, other heuristics could potentially be used by participants, such as the running average of *all* previous trials in the experiment as a prior [29], which could be explored in future studies.

## 5. Conclusions

Our results showed that participants’ performance predictions (before receiving the neurofeedback) improved throughout training, beyond what was explained by a potential heuristic based on previous performance. However, the absolute levels of confidence did not change, and the trial-by-trial confidence did not differentiate between the better and worse predictions either. In summary, our study unveils a dissociation between the cognitive factors affecting performance predictions and confidence levels, suggesting avenues for further investigation into their relationship.

## Data Availability

Experimental materials, analysis scripts and behavioural data (including online neurofeedback values) have been made available on a permanent archive [58]. Supplementary material is available online [59].
